# Oncolytic Viruses for Canine Cancer Treatment

**DOI:** 10.3390/cancers10110404

**Published:** 2018-10-27

**Authors:** Diana Sánchez, Gabriela Cesarman-Maus, Alfredo Amador-Molina, Marcela Lizano

**Affiliations:** 1 Unidad de Investigación Biomédica en Cáncer, Instituto Nacional de Cancerología-Instituto de Investigaciones Biomédicas, Universidad Nacional Autónoma de México, Mexico City 14080, Mexico; dianasanchezc@hotmail.com (D.S.); aamadorm@incan.edu.mx (A.A.-M.); 2 Department of Hematology, Instituto Nacional de Cancerología, Mexico City 14080, Mexico; gcesarman@gmail.com

**Keywords:** oncolytic virus, canine cancer, immunotherapy, canine treatment

## Abstract

Oncolytic virotherapy has been investigated for several decades and is emerging as a plausible biological therapy with several ongoing clinical trials and two viruses are now approved for cancer treatment in humans. The direct cytotoxicity and immune-stimulatory effects make oncolytic viruses an interesting strategy for cancer treatment. In this review, we summarize the results of in vitro and in vivo published studies of oncolytic viruses in different phases of evaluation in dogs, using PubMed and Google scholar as search platforms, without time restrictions (to date). Natural and genetically modified oncolytic viruses were evaluated with some encouraging results. The most studied viruses to date are the reovirus, myxoma virus, and vaccinia, tested mostly in solid tumors such as osteosarcomas, mammary gland tumors, soft tissue sarcomas, and mastocytomas. Although the results are promising, there are issues that need addressing such as ensuring tumor specificity, developing optimal dosing, circumventing preexisting antibodies from previous exposure or the development of antibodies during treatment, and assuring a reasonable safety profile, all of which are required in order to make this approach a successful therapy in dogs.

## 1. Introduction

The concept of treatment in cancer changed radically in recent years, with modulation of the immune response to tumor cells now shown to be an effective therapeutic strategy for achieving response in a growing number of cancer types [1]. This new stage in medicine was brought about by the first positive results in humans with solid tumors such as non-small-cell lung cancer, kidney cancer, and melanoma reporting prolonged survival, despite advanced disease, with the use of immune checkpoint inhibitors [2,3,4]. Oncolytic viruses, aside from displaying selective cytotoxicity toward cancer cells, may favor the restoration of the immune anti-cancer function [5,6]. This dual effect makes them an interesting strategy for the treatment of cancer, not only for humans but also for dogs.

Canine cancer is an increasing cause of death worldwide, principally in adult dogs, with an annual incidence rate of malignant tumors of about eight cases per 1000 dogs [7,8,9,10,11]. Among the most common types of cancers are lymphomas [8], hemangiosarcomas [12], mast cell tumors [13], melanomas [14], osteosarcomas [15], and mammary gland tumors [16]. After the introduction of chemotherapy for dogs in 1946 [17], different types of cancers became treatable with some prolonged clinical remissions using multidrug protocols [18]. However, although treatments evolved, complete long-lasting remissions in non-surgically curable tumors are still infrequent [19,20]. Importantly, the biological, genetic, phenotypic, and clinical similarities between dogs and humans allow comparative approaches favoring a faster clinical application of the research findings for both species [21,22,23].

## 2. Brief History of Oncolytic Viruses

Oncolytic viruses have a natural or acquired selectivity for cancer cells. Wild-type, attenuated, and genetically engineered viruses with varying anti-tumoral efficacies were studied [24,25,26]. From the beginning of the 20th century, case reports of tumor responses following a natural viral infection in people with cancer were published [27,28,29,30]. Preclinical studies in murine models showed tumor responses using viruses, and, by the 1950s, results from the first clinical trials in humans were reported with occasional responses, such as those using the Harries rabies vaccine with a response in 2/12 patients with melanoma, and the Egypt 10 virus in 4/34 patients with other solid tumors [31,32,33,34]. Clinical trials with oncolytic viruses are currently ongoing in humans and two viruses were approved for commercial use. The first, H101 or Oncorine^®^, is an adenoviral construct with an *E1B* deletion (in order to avoid replication in normal cells), approved in China in 2005 for the treatment of head-and-neck squamous cell carcinoma [35]. The second, OncoVex^GM-CSF^ or Imlygic^TM^, is an engineered herpes simplex virus type I (HSV-1) that expresses the human granulocyte–monocyte colony-stimulating factor as an immune-stimulant, approved in 2015 by the United States of America (USA) Food and Drug Administration (FDA) for the local treatment of unresectable cutaneous, subcutaneous, and nodal lesions in patients with recurrent melanoma after initial surgery [36]. In veterinary medicine, double- and single-stranded RNA and DNA viruses with natural oncolytic capacity, as well as genetically modified viruses, are being studied, still lagging behind human research. The initial results in dogs and humans are pushing forward the development of oncolytic viruses as exciting cancer treatment strategies. 

## 3. Oncolytic Viruses for Canine Cancer Treatment

**Figure 1:**
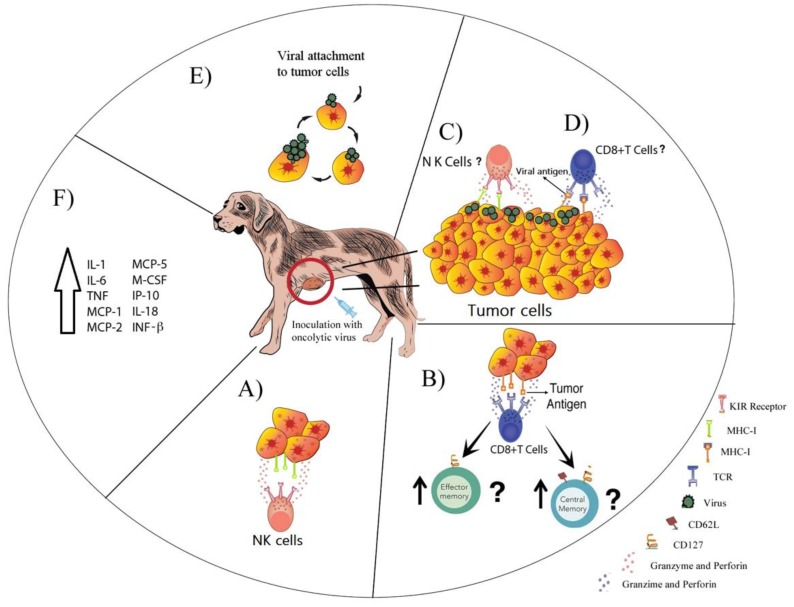
Possible antitumor mechanisms of oncolytic viruses. The possible mechanisms exerted by oncolytic viruses include the recognition and elimination of infected tumor cells by (**A**) natural killer (NK) cells or (**B**) cluster of differentiation 8 positive (CD8^+^) T cells, which can generate central and effector memory CD8^+^ T cells. It remains unknown if (**C**) NK cells and (**D**) CD8^+^ T cells can also eliminate uninfected tumor cells. (**E**) The oncolytic virus can also infect tumor cells and induce direct cell lysis. In addition, (**F**) inoculation with oncolytic viruses can enhance the secretion of several pro-inflammatory cytokines. Several pathways may occur simultaneously.

A total of 13 viral species were studied as oncolytics in dogs (Figure S1). The possible antitumor mechanisms are shown in Figure 1, and the findings of published data are described below with the information grouped by viral families. For information regarding each virus, we took data from the *Fields Virology* book into consideration, in addition to the references cited [37]. 

### 3.1. *Paramyxoviridae* Family

These viruses enter cells via fusion or receptor-mediated endocytosis [38,39,40]. As oncolytics, paramyxoviruses (Table 1) promote immune-mediated tumor cell death. Importantly, many of the receptors used by paramyxoviruses are over-expressed in cancer cells. Moreover, due to tumor-associated genetic defects in the interferon (IFN) and apoptotic pathways, which may be seen in cancer cells, viral replication occurs naturally and preferentially in malignant cells [41]. 

#### 3.1.1. *Measles Virus* (MV)

The oncolytic potential of MV was first observed in anecdotal reports describing the regression of hematopoietic neoplasms after accidental viral infection [42,43]. The first MV studied was the Edmonston-B vaccine strain, which is used worldwide for immunization against measles [44,45]. MV naturally infects lymphoid and respiratory epithelial cells. The infection of lymphoid cells is mediated by the binding of the H viral protein to CD150 (SLAM: signaling lymphocytic activation molecule) and to CD46 (membrane complement regulatory protein). In respiratory epithelial cells, MV binds through the nectin-4 receptor [46,47]. Nectin-4 is a receptor found to be overexpressed in some human cancers such as ovary, breast, and lung [48,49,50]. The MV genetic variant rMV-SLAMblind, which uses the related poliovirus 4 receptor (PVRL4/nectin-4) to enter cancer cells, was tested against human breast cancer with efficacy in xenografted mice and no relevant toxicity in primates [51]. Given the similarities between human breast cancer and canine mammary gland tumors (MGT), the virus was tested in canine MGT cells in vitro. The rMV-SLAMblind variant induced cell death in a manner dependent on the positive expression of canine nectin-4. Furthermore, immunosuppressed mice xenografted with canine nectin-4-positive MGT cells showed at least a 50% decrease in tumor volume compared to controls [52]. This therapy would make sense for dogs in the metastatic setting or in non-surgically resectable tumors, given that primary MGTs are usually well controlled with surgery [53]. Neurotoxicity still needs to be addressed and avoided [54]. In contrast to humans, the lack of pre-existing neutralizing antibodies against MV in dogs could favor in a systemic approach. 

#### 3.1.2. *Canine Distemper Virus* (CDV)

During a natural infection, CDV replicates in the epithelial cells of the respiratory tract [55]. The infection in dogs is clinically associated with respiratory and/or nervous system symptoms, in addition to severe long-term lymphoid depletion and immunosuppression [56,57]. Similar to measles, CDV employs CD150 and nectin-4 as cellular receptors [54,58]. In fact, central nervous system infection was associated with the expression of the neuronal nectin-4 receptor, present in dogs but not in humans [54].

In vitro, CDV was able to kill human cervical cancer cells [59] and acute T-cell lymphoblastic leukemia blasts [60]. In dogs, CDV was tested in histiocytic sarcoma and malignant lymphoid cells. The Onderstepoort strain of CDV infected and killed histiocytic sarcoma cells in vitro, inducing the expression of pro-inflammatory cytokines interleukin-1 (IL-1), (IL-6), and tumor necrosis factor (TNF) [61]. FXNO, YSA-TC, and MD-77 strains were also able to infect canine histiocytic sarcoma cells, with FXNO producing a more prominent and early cytopathic effect [62]. A recombinant strain, pCDVeGFPΔN, was able to infect canine malignant lymphoid cell lines, causing substantial cell death via apoptosis. An efficient infection with pCDVeGFPΔN strain was also demonstrated in primary canine malignant lymphocytes of B- and T-cell origin, although its oncolytic efficacy in those cells could not be demonstrated [60]. The possible inhibitory activity of morbilliviruses on cancer cells could be associated with the inhibition of metalloproteinases (MMPs), since persistent infection of the Onderstepoort strain in tumor cells derived from a malignant histiocytosis restored the activity of an MMP inhibitor decreasing the expression of MMPs [63]. These in vitro results are encouraging, because there is currently no effective established therapy for canine histiocytic sarcoma [64]. 

CDV was evaluated in seven dogs with lymphoma. The virus was injected intratumorally (IT) either in single or multiple doses of 1 × 10^6^ plaque-forming units (PFU) showing a low-toxicity profile with an intense fibrotic reaction in the injected lymph nodes. The CDV antigen was variably positive determined using immunohistochemistry in the treated lymph nodes, and, using co-cultures, the virus could only be isolated from treated nodes, but neither from distant lymph nodes nor from peripheral blood mononuclear cells (PBMCs). A strong anti-CDV antibody response was documented, ruling out the possibility of repeated dosing [65]. Furthermore, CDV is part of routine vaccination in dogs; thus, pre-existing antibodies are also a limitation for oncolytic treatment [66].

#### 3.1.3. *Newcastle Disease Virus* (NDV)

NDV naturally infects birds and, although it is considered a zoonosis, in humans, only self-limiting conjunctivitis was reported [67]. This virus showed natural oncolytic effects, principally in cells derived from human solid tumors [68,69,70]. A lentogenic strain was evaluated in canine cancer cells in vitro and in a dog with a spontaneous cancer [71,72]. In vitro, the NDV-MLS strain was preferentially toxic for canine malignant cells versus healthy PBMCs. The virus decreased the viability of primary canine B lymphoma cells by close to 40% [72]. In this same study, the early viral biodistribution was evaluated in a dog with a spontaneous T-cell lymphoma, 24 h after the intravenous (IV) administration of 1 × 10^12^ median tissue culture infectious dose (TCID_50_). Viral dissemination was detected by PCR only in healthy kidney, salivary gland, lung, and stomach, but not in tumor tissue, with no abnormalities upon histopathological assessment [72]. Interestingly, a complete long-term response in a dog with an aggressive chemotherapy-unresponsive lymphoma was reported [71].

It is probable that not all lymphomas are equally sensitive to NDV, and factors such as dose and route of administration influence the appropriate delivery of the virus to the tumor tissue. Furthermore, the historic safety profile of non-virulent strains [67] and immune-stimulatory potential [73,74,75] encourage the study of NDV for both human and canine cancers, with emphasis on its possible immune-stimulatory benefit. 

#### 3.1.4. *Sendai Virus* (SV)

This virus, also known as hemagglutinating virus of Japan, is a restricted pathogen in rodents, producing transmissible respiratory tract infections. SV was evaluated in vitro and in vivo as an oncolytic for human cancers, mainly in colon carcinoma, melanoma, and glioblastoma [76,77,78]. In one study, SV was evaluated in vivo in six dogs as therapy for mastocytoma. Dogs were treated with 1 × 10^7^ to 1 × 10^8.6^ mean embryo infectious dose (EID_50_) given in multiple injections (intratumorally and intradermally surrounding the tumor). Two or more viral treatments at one- or two-week intervals were given alone or in combination with surgery. Side effects included slight tenderness and edema at the injection sites which were responsive to antihistamines with quick resolution. Four dogs with either primary or recurrent tumors achieved complete responses, three of them long-lasting. The remaining two achieved stable disease [79]. These results justify further clinical studies of SV for the treatment of canine cutaneous/subcutaneous mastocytoma. Small lesions showed a better response to SV therapy [79]; yet, in routine practice, these lesions are usually resected. [80]. It would be interesting to test the use of this virus in an adjuvant or neo-adjuvant setting for mastocytoma, in order to determine whether the virus is able to control residual disease or reduce tumor burden facilitating surgery. It also will be important to evaluate drug interactions, especially when combined with steroids, which could decrease its therapeutic efficacy [79]. Following a pathological exam, SV was shown to induce infiltration by dendritic cells, CD4 and CD8 T-cells, and natural killer (NK) cells [77,81]. 

### 3.2. *Rabdoviridae Family*

These viruses enter cells via clathrin-mediated endocytosis [82], and the only species studied thus far as an oncolytic is the *vesicular stomatitis virus* (VSV). 

#### *Vesicular Stomatitis Virus* 

VSV causes an important endemic disease in cattle, while humans can be infected, and little is known about the dog as a host [83,84]. Although VSV has a natural oncolytic ability, genetic variants were created to improve therapeutic safety. The VSV-IFNβ-NIS is a variant expressing the human IFN-β (interferon beta) and the sodium iodide symporter (NIS). IFN-β exerts a protective effect on healthy cells mediated by IFN, while the expression of NIS makes molecular imaging of viral spread with radioactive iodine possible [85]. VSV-IFNβ-NIS is cytotoxic for human leukemic cells in vitro and for human myeloma in murine models [86]. Regarding dogs, two studies were performed using this virus. In a safety study in healthy Beagles treated with escalating doses starting at 1 × 10^8^ TCID_50_, no viral particles (v.p.) were detected in urine, saliva, or feces, and the intracranial and IV administration did not result in neurotoxicity [85]. Moreover, when 10 dogs with hematological and solid tumors were treated with 1 × 10^10^ TCID_50_/0.5 m^2^ IV, two short-lasting partial responses were observed in T-cell lymphoma with the longest response corresponding to a dog with sustained expression of IFN-β and higher number of viral copies in blood. Tumor infiltration by T cells was also documented in a case of anal adenocarcinoma. Regarding toxicity and environmental safety, all dogs developed mild short-lasting fever, as well as mild transient lymphopenia; two dogs developed transient hepatotoxicity, and there were no documented adverse events and no viral shedding in a dog with osteosarcoma (OSA) who received two viral doses [87]. Studies of VSV-IFNβ-NIS in dogs are a clear example of the benefit of dogs as preclinical models for humans. Regarding safety, neither humans nor dogs develop neurotoxicity [85,87], while, in rats and mice, neurotoxicity with seizures was seen within the first week post-treatment [88]. In fact, based on clinical responses to VSV-IFNβ-NIS in canine lymphoma patients, this VSV variant is currently being tested in a clinical trial for human patients with relapsed or refractory myeloid and lymphoid malignancies (clinicaltrials.gov identifier: MCT03017820). 

Another VSV variant tested as an oncolytic is VSV-GP, in which the VSV glycoprotein G was substituted, inducing less neurotoxicity [89]. VSV-GP is able to lyse melanoma cells in vitro by 0.1 multiplicity of infection (MOI) of VSV-GP. VSV-GP was also shown to prolong survival in mouse melanoma models (human xenografts and syngeneic) when treated with two doses of 10^7^ PFU, with a median survival of 45 vs. 22 days between treated and control mice, respectively. However, even though tumor growth was delayed, only few animals achieved long-term tumor remissions. [90]. The induction of type I interferon may have compromised the clinical efficacy due to the inhibition of viral replication. Interestingly, this may be overcome by inhibiting downstream signaling of the INF-receptor with the use of ruxolitinib, an inhibitor of Janus kinases 1/2 (JAK ½). These results suggest the possibility of using VSV-GP for canine melanoma and possibly for other tumors [90]. 

### 3.3. *Togaviridae Family*

This family includes the alphavirus genera. As oncolytic viruses, alphaviruses are non-virulent, remain non-pathogenic in healthy tissue, and are spontaneously eliminated by the immune system [91]. The majority of alphaviruses show neurotropism and are able to cross the blood–brain barrier [92]. The Semliki Forest virus is the only alphavirus that was tested as an oncolytic for canine cancer.

#### *Semliki Forest Virus* (SFV)

SFV can naturally infect humans and other animals causing neurological manifestations [93,94,95]. However, no natural infections were described in dogs, and viral injection into puppies did not cause neurological toxicity [96]. As an oncolytic, the VA7 attenuated strain was able to eradicate xenografts of human brain tumors and melanoma in immunocompromised mice [97,98]. The oncolytic capacity of attenuated SFV was shown to depend on the loss of IFN-I response in the tumor cells [99]. In the case of dogs, the VA7 strain expressing the enhanced green fluorescent protein (eGFP) caused progressive cytopathic effects and cell death in canine OSA cells in vitro. In addition, injection of approximately 2 × 10^5^ PFU IV of SFV in two laboratory Beagles did not result in signs of infection or severe adverse events. The neutralizing antibody response indicated that both dogs mounted a humoral immune response in the absence of detectable viremia; there were no histopathological changes in injected tissues, and no viral antigen was detected through immunohistochemistry in any of the organs analyzed. Regarding environmental safety, the virus was not recovered from serum, urine, or feces [100]. Given the safety profile and the positive result obtained in human melanoma xenografts in mice [97], as well as the limited effective treatments available for canine melanoma [101], the study of SFV should be further pursued. 

### 3.4. *Reoviridae Family*

Reoviruses have the ability to infect almost all known mammal species and cause mild physical illness in humans [102]. Anti-reovirus neutralizing antibodies are detected in almost all adult humans and in a high percentage of healthy dogs [103,104,105]. Despite this, several strains of reoviruses were evaluated as oncolytics in humans; however, only reovirus-3, naturally isolated from dogs with diarrhea, was tested in dogs [106]. 

#### *Reovirus* Serotype 3 

Dearing is the most used oncolytic strain (Reolysin^®^, from Oncolytics^TM^ Biotech Inc., Calgary, AB, Canada) to date. In humans, Reolysin^®^ was tested in phase II and III clinical trials [106]. In dogs, Reolysin^®^ showed potential in vitro for the treatment of mastocytoma (MCT), lymphoma, MGT, and melanoma cells. Canine visceral and cutaneous MCT cells were efficiently killed by Reolysin^®^ via apoptosis. In addition, mice xenografted with visceral canine MCT experienced significant tumor regression after a single treatment (*p* < 0.05), and, when one of the tumors was intratumorally injected in a bilateral tumor model, both tumors showed significant regression with extensive necrotic lesions. This was associated with an adequate generation of progeny and hematogenous spread. However, Reolysin has low specificity for malignant mastocytes, with nearly 90% death of normal canine bone marrow mast cells in vitro [107]. Induction of apoptosis and a significant decrease in cell viability were also documented in both T- and B-cell lymphomas in vitro, and mice xenografted with canine T-cell lymphoma treated with 1 × 10^8^ PFU IT showed significant suppression of tumor growth compared to those treated with ultraviolet (UV)-inactivated reovirus (*p* < 0.05) [25]. A similar in vitro effect was demonstrated in canine OSA, MGT, and melanoma cell lines, where Reolysin^®^ reduced cell viability through caspase-3-mediated apoptosis. The production of viral progeny was detected in susceptible lines. Susceptibility of human cancer cells to reovirus infection was associated with the activation of the Ras signaling pathway [108]; yet, interestingly, oncolysis by reoviruses in canine cancer cells from MCT, lymphoma, OSA, MGT, and melanomas does not seem to depend on the state of Ras activation [25,107,109]. 

Importantly, the safety profile of Reolysin^®^ was established in 19 dogs with advanced cancer. Most of the patients had MCT, lymphoma, oral melanoma, or soft tissue sarcoma (STS). The dogs received doses that varied from 1 × 10^8^ to 5 × 10^9^ TCID_50_ via either IT injection (10 dogs) or IV injection (nine dogs) daily for five consecutive days, for one or multiple treatment cycles. Live virus in serum was detected only in one dog during the first cycle of chemotherapy, but not in other cycles. All dogs experienced an increase in the anti-reovirus neutralizing antibody titer, and there was a reduction in the size of the lesions in five dogs; in six others, there was a benefit in clinical signs (improvement of urination, reduction of tumor pain, increased mobility, or improvement in appetite) [110]. Thus, there was a good safety profile, as well as tumor response, in a subset of dogs [110]. The significance of the presence of neutralizing antibodies prior to therapy and the effect on clinical response from increasing anti-reovirus neutralizing antibody titers with therapy need to be studied in dogs. Considering the experience observed in humans [111,112,113], a combined approach with chemotherapy, radiotherapy, or other therapies could be explored in dogs. 

### 3.5. *Parvoviridae Family*

Parvoviruses are the smallest viruses in existence [114]. This family includes the mouse minute virus (MVM), the rat H-1PV virus, the LuIII virus, and canine parvovirus (CPV), which showed natural oncolytic potential for human cancers cells in vitro and/or in vivo [115,116,117,118,119]. CPV was genetically engineered for cancer therapy [120], and studies related to its life cycle were investigated and provided insights into its use as an oncolytic for dogs [119]. 

#### *Canine Parvovirus* Serotype 2 (CPV-2)

CPV-2 is an important pathogen in domestic and wild canids; it can cause acute hemorrhagic enteritis and myocarditis, and it is commonly fatal [121,122,123,124]. However, vaccination can provide effective protection [125,126,127]. During natural infections, CPV-2 binds to the transferrin receptor (TfR), followed by clathrin-mediated endocytosis [128]. In vitro, CPV-2 induces cell death of canine fibroma cells, characterized by apoptosis and subsequent necrosis [119].

Although benign, the infiltrative ability of fibrous proliferations, depending on the anatomical site, makes surgical procedures difficult and/or invasive [129,130,131,132,133]. CDV could represent an alternative option for these patients. The virus could be explored for canine cancer, and inflammation secondary to necrosis could be beneficial in oncolytic virotherapy, since inflammation associated with necrosis might favor the activation of the immune system [134].

### 3.6. *Poxviridae Family*

Poxviruses enter cells via fusion and replicate predominantly in the cytoplasm [135]. In dogs, three genera of poxviruses were studied as oncolytics (Table 2). 

#### 3.6.1. *Myxoma Virus* (MYXV)

MYXV is only pathogenic for rabbits causing myxomatosis, a lethal disease [144]. As an oncolytic, MYXV is able to kill different types of human cancer cells in vitro (pancreas, ovary, glioblastoma, and hematological neoplasms) [145,146,147,148]. The greater susceptibility of these cells to MYXV was associated with the increased expression of phosphorylated protein kinase B (pAkt) [149]. MYXV and a MYXVΔserp1, which is a less pathogenic MYXV strain for rabbits [150], were evaluated in canine cancer cells. MYXVΔserp1 lost the expression of serp2, an anti-apoptotic virulence factor. At an MOI of 5, this virus was able to significantly kill hemangiosarcoma (HSA) and transitional cell carcinoma (TCC) cell lines, as well as primary HSA and STS cells. Compared to human cancer cells, MYXV was not as effective at killing canine cancer derived from dogs [151]. MYXVΔserp1 was studied in 10 dogs with STS, with either a single IT dose of 1 × 10^6^ PFU or post-operatively (5–10 doses of 1 × 10^6^ PFU) in incompletely excised tumors (positive surgical margins) in an attempt to control residual disease. Although the study demonstrated clinical and environmental safety parameters, the majority of patients treated intratumorally (4/5) only achieved stable disease and one progressed after a moth despite treatment. The report of outcome in post-surgically treated patients [136] and data on whether the virus can delay or decrease metastasis are still not available. Metastasis is another important complication in canine patients with high-grade STS [152]. Additionally, research on MYXV as an oncolytic for dogs should also consider the effectiveness related to pAkt expression, which would suggest that tumors such as canine melanoma and mastocytoma which expresses pAkt [153,154] may become possible targets.

#### 3.6.2. *Canarypox Virus*

This is a virus of the genus *Avipoxvirus*. Avipoxviruses only infect some mammalian cells under laboratory conditions [155,156] and, although human healthy cells can be readily infected, the full replication cycle does not occur. However, a genetic variant (ALVAC) derived from the Kanapox strain of the canarypox virus was developed in order to address safety concerns. This variant is under investigation for its stimulatory properties on peripheral cytokine and chemokine response, and for possible use as therapy for human cancer [157]. When ALVAC-luciferase was administered IT in a dog with melanoma, the virus demonstrated limited viral dissemination; it was localized only along the needle path with no detectable virus in the periphery of the tumor [158]. This restricted dissemination could represent a safety advantage for dogs as was the case of ALVAC-derived recombinants that express the stimulatory cytokine IL-2, which were used successfully in the control of recurrent feline fibrosarcoma [158]. 

#### 3.6.3. *Vaccinia Virus* (VACV) 

VACV is widely used as a vaccine agent against smallpox [137], and diverse genetically engineered strains showed oncolytic potential [159,160]. Five VACVs derived from the Lister strain and one derived from the Copenhagen strain were evaluated as oncolytics for canine cancers. GLV-1h68 (GL-ONC1 from Genelux Corporation), a Lister-derived strain which encodes a light-emitting fusion gene, β-galactosidase, and β-glucuronidase, was first evaluated in a human breast xenograft mouse model, where a single dose was capable of causing complete tumor regression [161]. Furthermore, in in vitro studies, GLV-1h68 induced viral replication-dependent apoptosis of canine mammary adenoma cells [162], while, in canine mammary carcinoma, GLV-1h68 was able to efficiently infect, replicate, and lyse cells [138]. In murine models, GLV-1h68 induced significant tumor regression of canine adenoma xenografts [162], and a single IV dose of 5 × 10^6^ PFU in nude mice xenografted with mammary carcinoma induced a strong inflammatory and oncolytic response that resulted in significant inhibition of tumor growth with confirmed infection and replication capability and tumor specificity [138]. GLV-1h68 also enhanced the expression of monocyte chemo-attractant protein (MCP)-1, -2, and -5, macrophage colony stimulating factor (M-CSF), interferon gamma-induced protein (IP)-10, and IL-18, which are known to increase innate immunity mediated by dendritic cells, neutrophils, and macrophages. This suggests that the activation of the innate immune system could act along with viral oncolysis to induce inhibition and tumor clearance in the murine model of canine mammary carcinoma [138]. 

Another VACV, LIVP1.1.1, was isolated from a wild-type stock of Lister strain. LIVP1.1.1, together with GLV-1h68, was evaluated in vitro and in vivo in a murine model of canine STS [139]. Both strains were shown to infect and destroy STS cells in vitro with better replication efficiency by LIVP1.1.1 [139]. Mice with canine xenografts of STS, treated IV with 1 × 10^7^ PFU of LIVP1.1.1 or GLV-1h68, had reduced tumor growth [139]. The virus induced tumor necrosis and an immune infiltrate, as well as CD31 overexpression, which suggests that endothelial activation could be an additional reason for the increased accumulation of immune cells in the tumor site of the treated mice [139]. 

The GLV-1h109 strain is derived from GLV-1h68, but contains the *GLAF-1* gene, which encodes for single-chain anti-vascular endothelial growth factor (anti-VEGF) antibody as a strategy to regulate tumor angiogenesis. Although anti-VEGF is directed against human and murine VEGF, it also recognizes canine VEGF [140]. In in vitro studies, GLV-1h109 infected, replicated, and lysed canine STS and prostatic carcinoma cells, decreasing viability by more than 70% [140]. In in vivo murine models, GLV-1h109 showed improved tumor-specific replication as compared to GLV-1h68. Treatment with GLV-1h109 inhibited the growth of murine xenografts of canine prostatic carcinoma and canine STS with acceptable toxicity. In these models, tumors infected with GLV-1h109 showed increased accumulation of neutrophils, myeloid-derived suppressor cells, and macrophages, as well as a reduction in blood vessel density in STS [140]. 

Another oncolytic strain is LIVP6.1.1, which was also isolated from a stock of wild-type Lister strain; it is less virulent as compared to other isolates. LIVP6.1.1 was evaluated in canine STS, prostate carcinoma, melanoma, and OSA [141]. In in vitro experiments, infection by LIVP6.1.1 efficiently killed the four cell types mentioned, causing at least 83% cytotoxicity [141]. In in vivo experiments in mice xenografted with canine STS or prostate carcinoma and treated with an IV dose of 5 × 10^6^ PFU, there was a significant decrease in tumor growth (close to 50%), without signs of toxicity [141]. Interestingly, LIVP6.1.1 also showed preference for replication at the tumor site. In addition, an increase in immune cell infiltrate (granulocytes, monocytes, macrophages, and major histocompatibility complex class II positive (MHCII^+^) CD45^+^ cells) was documented in STS tumors [141]. In peripheral blood, no changes were found in cell populations, suggesting that response to virotherapy with LIVP6.1.1 in xenografts could be a combined result of direct oncolysis in addition to tumor-localized immune stimulation [141]. 

GLV-5b451 derived from the LIVP 6.1.1 strain contains the *GLAF-1* gene encoding for a single chain of anti-canine VEGF antibody [142]. In dogs, VEGF overexpression was correlated with tumor malignancy and poor prognosis [143,163,164,165,166]. In in vitro studies, GLV-5b451 effectively infected, replicated, and killed canine cells from mammary carcinoma, mammary adenoma, prostatic carcinoma, and STS, inducing at least 60% cell death. These four cell types produce VEGF, especially prostatic carcinoma [142]. In a murine model of canine STS, a single dose of 1 × 10^7^ PFU of GLV-5b541 or LIVP 6.1.1 was able to inhibit tumor growth without evidence of toxicity. GLV-5b541 showed greater efficacy and led to significant inhibition of tumor blood vessel development and increased expression of CD31 compared to LIVP 6.1.1. The upregulation of CD31 protein could mediate the trans-endothelial migration of immune cells to the site of infection. In this model, viral replication was preferentially seen in tumors compared to healthy tissues such as spleen, liver, and lungs [142]. 

Finally, NYVAC, derived from the Copenhagen strain, was first tested in healthy dogs. After parenteral (oral submucosa) administration, NYVAC-luciferase was recovered from the site of inoculation within 10 min, after which it was no longer detectable. Importantly, the virus was not detectable in saliva, feces, or blood at any time [158]. In addition, a dog with spontaneous melanoma treated IT with NYVAC showed localized dissemination only to the site of injection restricted to the needle path [158]. Viruses derived from the Lister strain of VACV seem to be promising agents for canine cancer immunotherapy. Studies of immune response in immune-competent animals are still warranted.

### 3.7. *Adenoviridae Family*

Adenoviruses naturally infect a great diversity of animals, including dogs and humans [167,168]; however, their tropism can be modified to direct the infection toward specific cell types [169]. Adenoviruses modified to selectively replicate in cancer cells and to induce cytolysis are termed “conditionally replicating oncolytic adenoviruses” (CRAds) [170]. CRAds based on human adenovirus 5 and canine adenovirus 2 were evaluated as oncolytics for canine cancer.

#### 3.7.1. *Canine Adenovirus* Type 2 (CAV-2) 

This virus causes a respiratory disease in dogs characterized mainly by bronchitis and bronchiolitis. It was used to immunize dogs against the related virus CAV-1, associated with highly contagious hepatitis in dogs [125]. 

The CAV-2-based vectors, OC-CAVE1 and ICOCAV17, were evaluated as oncolytics for canine cancer. OC-CAVE1 contains the osteocalcin (OC) promoter and, since tumors such as osteosarcoma (OSA) have high osteocalcin activity, this property allows targeting viral replication to these tumor cells. Treatment with OC-CAVE1 in a murine model xenografted with canine OSA showed a significant (*p* < 0.005) anti-tumor effect [171]. In healthy dogs treated IV with 2 × 10^12^ v.p., there were no clinical signs of infection and no macroscopic or microscopic changes upon pathological examination, although viral DNA was detected at high levels in the spleen and liver. Of note, there is a safety concern given that viral DNA was detected in blood for at least 48 h and for more than 96 h in urine and feces [172]. Another concern is that, since the presence of neutralizing antibodies develops as a consequence of previous infections or vaccination programs, those antibodies could interfere with viral anti-cancer response. To overcome this issue, the use of canine cancer cells as carriers of the virus was evaluated. Canine OSA cells as carriers of OC-CAVE1 were tested in canine xenografts in mice; they successfully reached the tumor site after IV administration, with reduction of viral hepatic uptake and with effective control of tumor growth despite the presence of anti-CAV neutralizing antibodies [173]. Additionally, when a polylysine was added to OC-CAVE1, its infectivity improved, as demonstrated in primary and immortalized canine OSA cells in vitro [174]. 

ICOCAV17 expresses hyaluronidase, an enzyme that dissociates the extracellular matrix and, therefore, enhances viral distribution after IT administration [175]. This virus showed functional activity in canine OSA and melanoma cells in vitro, while mice with canine melanoma or canine OSA xenografts treated with three weekly IT doses of 1 × 10^10^ v.p. showed increased survival without signs of toxicity [175]. In addition, six dogs with different spontaneous cancers (adenoma, mastocytoma, fibrosarcoma, metastatic neuroendocrine tumor, and osteosarcoma) were treated with 1 × 10^12^ v.p. and did not show adverse effects, even with viral titers in serum reaching 1 × 10^6^ v.p./mL. Four of the six dogs responded to treatment, two with a partial and two with stable disease, and none of the six developed tumor lysis syndrome. Viral treatment was given concomitantly with the tyrosine kinase inhibitor, toceranib, in two of these patients. The virus caused tumor necrosis, and the viral genome could be detected in blood up to a week after treatment, with no viral shedding in urine, saliva, or feces at any time point [175]. Interestingly, this virus was used to infect canine mesenchymal stem cells, which were then infused intravenously as carriers for the virus [176]. There were no significant adverse effects in the 27 patients treated, with an overall response of 74% and 14.8% complete response rate, including remission of lung metastasis. Additionally, the virus induced inflammatory cell infiltration observed in tumor biopsies after four weeks of treatment. Apparently, the development of antibodies did not interfere with clinical benefit [176]. 

#### 3.7.2. *Human Adenovirus* Type 5 (Ad5)

Ad5 causes infections of the respiratory tract in humans and was the platform most used for the development of oncolytic CRAds. In dogs, two vectors based on Ad5 were studied as oncolytics: Ad5CMVGFP and AdCD40L. Ad5CMVGFP expresses green fluorescent protein and was shown to efficiently infect primary canine OSA cells in vitro, although at a higher MOI than required in cells of human origin [169]. 

AdCD40L codifies for human CD40L, a molecule activating dendritic cells and T helper 1 (Th1) lymphocytes. Human and canine CD40L have high homology (85%), which led to the study of AdCD40L in dogs. Initially, two dogs with malignant melanoma were treated with peritumoral and IT injections of 9.6 × 10^8^ v.p. One dog with a progressive conjunctival melanoma received six weekly treatments and, after initial inflammation, the tumor rapidly decreased in size; at four-month follow-up, there were no signs of progression or metastasis [177]. The other dog had an oral stage III melanoma and received two treatments with a four-day interval, achieving a long-term complete response. Interestingly, after viral treatment, the tumor tissue was infiltrated by both T and B lymphocytes. In both cases, the tumor lost pigmentation and there were no relevant signs of systemic autoimmunity [177]. It is important to mention that dogs with stage III melanoma treated only with surgery rarely survive more than 22 weeks [178]. AdCD40L was further evaluated in another 19 dogs with melanoma (14 oral, four cutaneous, and one conjunctival). The dogs received between one and six weekly IT doses with 37 µL of concentrated viral stock solution at 7 × 10^10^ PFU/mL at weekly intervals, followed by cytoreductive surgery in nine cases. Although there were mild adverse effects in 8/19 dogs, these did not recur with subsequent treatments. Additionally, there were five complete and eight partial documented responses, with four dogs being stable and two having progressive disease. Again, in 8/11 dogs, pathology showed tumor infiltration by B and T lymphocytes, suggesting local immune stimulation [179]. Oncolytic adenovirus-based vectors may represent an alternative option for the treatment of canine cancer. Mechanisms to circumvent the effect of pre-existing antibodies that could compromise effectiveness should be studied. 

## 4. Discussion

There is growing interest in the study of oncolytic viruses as an approach for the treatment of canine cancer. Table 3 summarizes the different studies performed to date in vitro and in vivo related to dogs. Most of the studies were performed in vitro, mainly with the use of reovirus, myxoma, and vaccinia, with encouraging results in tumor cells derived from osteosarcomas, mammary gland tumors, soft tissue sarcomas, and mastocytomas. There are very few studies addressing hematological malignancies; yet, some reports show promising oncolytic responses in canine lymphoma cells with the use of paramyxoviruses. Regarding in vivo studies in preclinical models (canine xenografts in mice), the control of tumor growth was consistently accompanied by virus-induced tumor infiltration by immune cells, as well as a decrease in tumor blood vessel density, and induction and over-expression of molecules involved in innate immunity, as seen with the vaccinia virus. It is important to note that dogs may better reflect the clinical efficacy and safety parameters associated with oncolytic virotherapy than mouse models, as seen with VSV. The published results strongly encourage continuing the study of oncolytic viruses using a comparative approach between dogs and humans.

## 5. Conclusions

Oncolytic viruses are gaining ground as an alternative approach for treating cancer in dogs and humans. Although most of the current information was obtained in vitro, there are encouraging in vivo results and growing interest in performing clinical studies. The tolerance and safety for viruses such as vesicular stomatitis, Semliki Forest, canarypox, and vaccinia were established in healthy dogs. Favorable clinical responses are being reported both with natural and genetically modified viruses in tumors such as lymphomas, melanomas, adenomas, and cutaneous mastocytomas. In order to improve the usefulness of oncolytic viruses for the treatment of tumors in dogs, several issues must be addressed. These include a better characterization of tumors, which still lags far behind that of studies in humans, as well as the search for canine cell-specific therapeutic targets, viral modifications to enhance tumor specificity, safety studies regarding viral shedding, the establishment of therapeutic regimens (doses and routes of administration), and the evaluation of combined therapies for improving tumor responses. These studies should lead, in the not too far future, to the use of oncolytic viruses as part of cancer treatment. 

## Figures and Tables

**Table 1:** Paramyxoviridae family and genera. Oncolytic paramyxoviruses are marked in bold.

Family	Genus	Virus
Paramyxoviridae	*Morbilivirus*	***Measles, canine distemper virus,*** *rinderpest, peste des petits ruminants*
	*Avulavirus*	***Newcastle disease virus***
	*Respirovirus*	***Sendai***
	*Rubulavirus*	Mumps *
	*Henipavirus*	*Hendra*
	*Aquaparamyxovirus*	*Salmon aquaparamyxovirus*
	*Ferlavirus*	*Reptilian ferlavirus*

* *Mumps virus* has a proven oncolytic effect in humans, but is yet to be tested in dogs.

**Table 2:** Poxviruses studied in canine tumors.

Family	Genus	Species	Virus	Tumor Type Studied	References
Poxviridae	*Leporipoxvirus*	*Myxoma virus*	MYXV wild-type	In vitro in osteosarcoma, transitional cell carcinoma, hemangiosarcoma, mastocytoma, soft tissue sarcoma, stromal gastrointestinal tumor, perianal adenoma, mixed mammary tumor, and renal carcinoma	[136]
			MYXVΔserp1		
	*Avipoxvirus*	*Canarypox virus*	ALVAC	Canine spontaneous melanoma	[137]
	*Ortopoxvirus*	*Vaccinia Virus*	GLV-1h68	Canine mammary adenoma and carcinoma, both in vitro and in vivo in mice	[138,139]
			LIVP1.1.1	Soft tissue sarcoma in vitro and in vivo in mice	[140]
			GLV-1h109	Soft tissue sarcoma in vitro and in vivo in mice, and in vitro in prostatic carcinoma	[141]
			LIVP6.1.1	Soft tissue sarcoma and prostatic carcinoma, both in vitro and in vivo in mice, and in vitro in melanoma and osteosarcoma	[142]
			GLV-5b451	In vitro in mammary carcinoma, mammary adenoma, and prostatic carcinoma, as well as in soft tissue sarcoma in vitro and in vivo in mice	[143]
			NYVAC	Canine spontaneous melanoma	[137]

**Table 3:** Oncolytic viral strains studied in dogs.

Genome	Family	Genus	Species	Strain Natural (N)/Modified (M)	Canine Model	References
ssRNA	Paramixoviridae	*Morbilivirus*	*Measles*	rMV-SLAMblind (M)	- Mammary gland tumor (Cell line CF33 nectine-4^+^)	[53]
			*Canine distemper virus* (CDV)	Onderstepoort (N)	- Histiocytic sarcoma (cell line DH82)	[62]
				FXNO (N), YSA-TC (N), and MD-77 (N)	- Histiocytic sarcoma (cell line CTT)	[63]
				pCDVeGFPΔN (M)	- Round-cell leukocytic neoplasia (cell line CCL-1390) - T-cell leukemia (cell line CLGL-90) - B-cell lymphoma (cell line 17-70)	[61]
				CDV (N)	- Canine lymphoma patient	[67]
		*Avulavirus*	*Newcastle disease virus* (NDV)	NDV-MLS (N)	- B-cell lymphoma (primary cells) - Healthy PBMC	[73]
		*Respirovirus*	*Sendai virus* (SV)	SV (N)	- Canine spontaneous mastocytoma	[80]
ssRNA	Rabdoviridae	*Vesiculovirus*	*Stomatitis vesicular virus* (VSV)	VSV-IFNβ-NIS (M)	- Healthy dogs - Canine cancer patients	[86,88]
ssRNA	Togaviridae	*Alfavirus*	*Semliki Forest virus*	VA7 (N)	- OSA (cell lines Abrams and D17) - Healthy dogs	[101]
dsRNA	Reoviridae	*Ortoreovirus*	*Reovirus*	Dearing (Reolysin^®^) (N)	- Visceral MCT (cell lines VIMC and CoMS) - Cutaneous MCT (cell lines CM-MC and HRMC) - T-cell lymphoma (cell lines CL-1, UL-1, CLGL-90, Nody-1, Ema, and CLK) - OSA (cell lines D17, Gracie, Abrams, MacKinley, and Moresco) - Mammary gland tumor (cell lines CIP-p, CHM-m, CNM-m, CIP-m, CTB-p, CTB-m, and CHM-p) - Melanoma (cell lines CMGD2, CMeC1, CMeC2, KMeC, and LMeC). - Advanced canine cancer patients	[25,108,109,111]
ssDNA	Parvoviridae	*Parvovirus*	*Canine parvovirus*	Canine parvovirus (N)	- Fibroma (cell line A27)	[120]
dsDNA	Poxviridae	*Leporipoxvirus*	*Mixoma virus* (MYXV)	MYXV (N)	- OSA (Abrams and D-17 cell lines) - TCC (Bliley cells) - HSA (Den and Fitz cells) - MCT (2 primary cell lines) - HSA (1 primary cell line) - STS (1 primary cell line) - Stromal gastrointestinal tumor (primary cells) - Perianal adenoma (cell line) - Mixed mammary tumor (cell line) - Renal carcinoma (primary cells) - Healthy fibrous tissue (cell line)	[151]
				MYXVΔserp1 (M)		
		*Avipoxvirus*	*Canaripox virus*	ALVAC (M)	- Canine spontaneous melanoma - Healthy dog	[137]
		*Ortopoxvirus*	*Vaccinia virus*	GLV-1h68 (M)	- Mammary adenoma (cell line ZMTH3 and canine xenograft in mouse) - Mammary carcinoma (cell line MTH52c and canine xenograft in mouse)	[138,139]
				LIVP1.1.1 (M)	- STS (cell line STSA-1 and canine xenograft in mouse)	[140]
				GLV-1h109 (M)	- STS (cell line STSA-1 and canine xenograft in mouse) - Prostatic carcinoma (cell line DTT08/40)	[141]
				LIVP6.1.1 (N)	- STS (cell line STSA-1 and xenograft in mouse) - Prostatic carcinoma (cell line DT08/40 and xenograft in mouse)- Melanoma (cell line CHAS) - OSA (cell line D17).	[142]
				GLV-5b451 (M)	- Mammary carcinoma (cell line MTH52c) - Mammary adenoma (cell line ZMTH3) - Prostatic carcinoma (cell line CT1258) - STS (cell line STSA-1 and xenograft in mouse)	[143]
				NYVAC (M)	- Canine melanoma patients - Healthy dog	[137]
dsDNA	Adenoviridae	*Mastadenovirus*	*Canine adenovirus* (CAV)	OC-CAVE1 (M)	- OSA (cell lines D22, D17, and CF11, and xenograft in mouse) - Healthy dogs	[172,173,174,175]
				ICOCAV17	- OSA (cell lines Abrams and D17) - Melanoma (cell lines CML1 and 17CM98) - Canine cancer patients	[176,177]
			*Human adenovirus* (Ad5)	Ad5CMVGFP (M)	- OSA (primary cells)	[170]
				AdCD40L (M)	- Canine malignant melanoma patients	[178,179]

ss: single-stranded; ds: double-stranded; PBMC: peripheral blood mononuclear cell; OSA: osteosarcoma; TCC: transitional cell carcinoma; HSA: hemangiosarcoma; MCT: mastocytoma; STS: soft tissue sarcoma.

## Supplementary Materials

The following are available online at http://www.mdpi.com/2072-6694/10/11/404/s1, Figure S1: Oncolytic viruses studied in dogs.
