# Supplementary material for: Oncolytic Viruses for Canine Cancer Treatment
Source: Cancers (Basel). 2018 Oct 27;10(11):404. doi: 10.3390/cancers10110404 (PMC6266482; doi:10.3390/cancers10110404)
Supplement: Supplementary file 1 [file cancers-10-00404-s001.pdf]

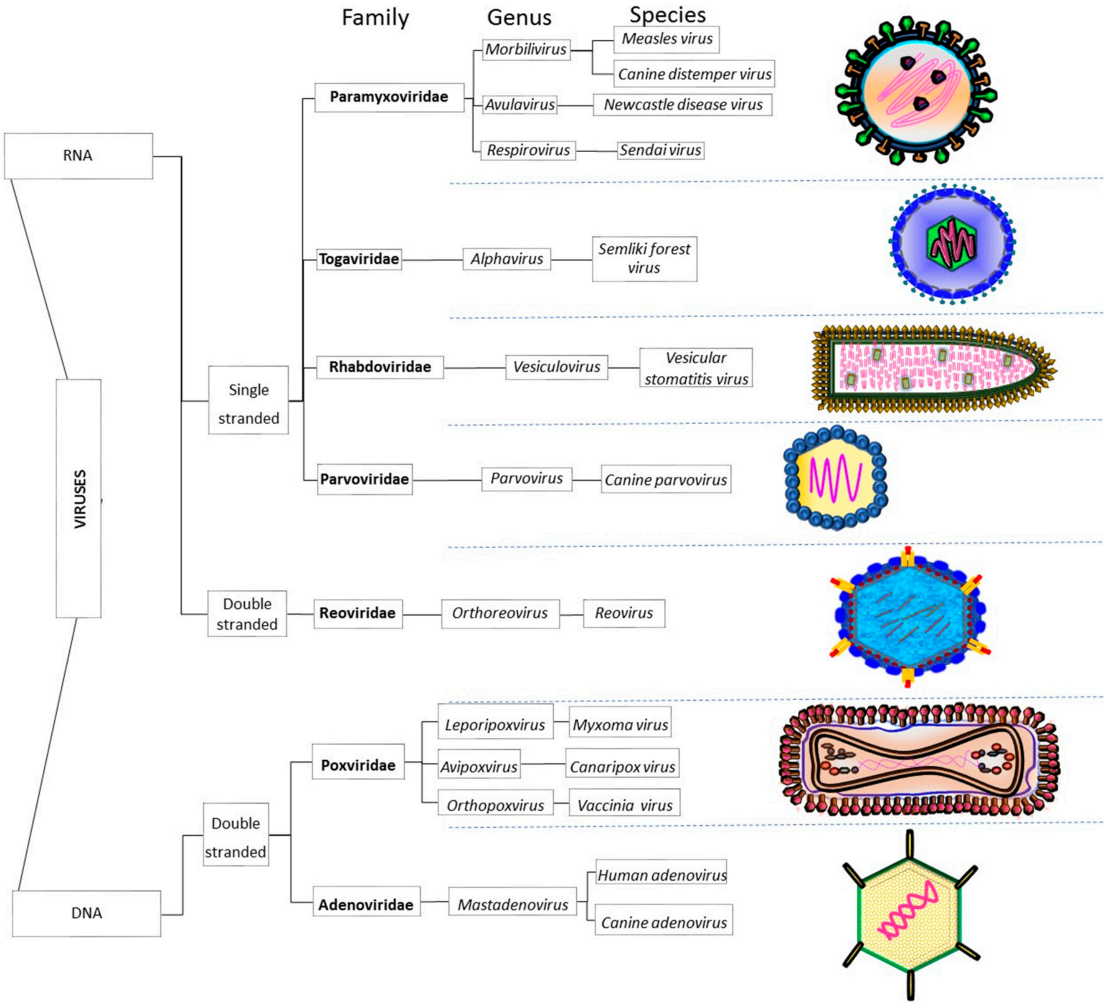

Supplementary Figure S1. Oncolytic viruses studied in dogs. The figure shows the different viruses that have been studied as oncolytics either *in vitro* or *in vivo* in dogs. Viruses are grouped according to their genus, family and type of nucleic acid. Representative virion structures for each viral family are shown.
